# A Practical Guide to Rodent Islet Isolation and Assessment

**DOI:** 10.1007/s12575-009-9021-0

**Published:** 2009-12-03

**Authors:** Jeffrey D Carter, Stacey B Dula, Kathryn L Corbin, Runpei Wu, Craig S Nunemaker

**Affiliations:** 1 Department of Medicine, Division of Endocrinology, University of Virginia, P.O. Box 801413, Charlottesville, VA, 22908, USA; 2 DERC Cell and Islet Isolation Core Facility, University of Virginia, Charlottesville, VA, USA

**Keywords:** islets of Langerhans, pancreatic islets, beta-cell, beta cells, cytokines, transplantation, calcium, insulin, apoptosis, necrosis, isolation, islet isolation, collagenase, rodent, mouse, rat, islet

## Abstract

Pancreatic islets of Langerhans secrete hormones that are vital to the regulation of blood glucose and are, therefore, a key focus of diabetes research. Purifying viable and functional islets from the pancreas for study is an intricate process. This review highlights the key elements involved with mouse and rat islet isolation, including choices of collagenase, the collagenase digestion process, purification of islets using a density gradient, and islet culture conditions. In addition, this paper reviews commonly used techniques for assessing islet viability and function, including visual assessment, fluorescent markers of cell death, glucose-stimulated insulin secretion, and intracellular calcium measurements. A detailed protocol is also included that describes a common method for rodent islet isolation that our laboratory uses to obtain viable and functional mouse islets for in vitro study of islet function, beta-cell physiology, and in vivo rodent islet transplantation. The purpose of this review is to serve as a resource and foundation for successfully procuring and purifying high-quality islets for research purposes.

## 1. Introduction

Pancreatic islets are thought to play a key role in the pathophysiology of Type 1 and Type 2 diabetes through the failure of islet beta cells to secrete sufficient quantities of insulin to regulate blood glucose [1]. In recent years, increasing interest surrounding islet replacement therapies in humans has provided the drive for advances in the methods used to isolate islets from humans as well as a host of animal research models [2,3]. Although there are many published islet isolation protocols specific to mouse and rat, few provide the necessary details for researchers to successfully perform the complex procedures.

The primary goal of isolating pancreatic islets, whether for in vivo transplantation or in vitro studies, is to obtain viable purified islets that respond in a manner consistent with their function in vivo. The key elements of a successful islet isolation procedure are: (1) enzymatically digesting the tissues connecting the islets to the exocrine tissue, (2) separating islets from non-islet tissue, and (3) culturing isolated islets in an environment that maintains cell viability. We review these key elements and provide methods for evaluating islet quality. In addition, we present a common method for the isolation of rodent islets used regularly in our islet isolation facility to consistently procure viable and functional islets for in vitro study of islet function, beta-cell physiology, and in vivo islet transplantation. Our protocol is, by no means, the only successful method for isolating pancreatic islets; however, the additional details provided in this protocol are intended to provide the rationale for each step in the process in order to assist researchers in their efforts to obtain healthy islets for study.

## 2. Procedures of islet isolation

### 2.1. Basic methodologies

The two most prevalent approaches for isolating islets from rodent pancreatic tissue differ, primarily, in the way digestive enzymes are introduced to the pancreatic tissue surrounding the islets. In the first approach, the pancreas is excised from a euthanized animal and cut into 1–2 mm pieces, thus increasing the surface area and providing conditions for the digestive enzyme collagenase to break down the tissue surrounding the islets [4,5]. The pancreatic pieces are enzymatically digested in a collagenase solution and concurrently mechanically digested by being stirred or shaken. In the second approach, described by Gotoh et al., collagenase is injected into the common bile duct (CBD) of a euthanized animal. The pancreas is then excised and digested at 37°C without being cut into pieces or mechanically digested [6].

Although these two approaches form the foundation for many islet isolation techniques, there are considerable variations in the details among published methods, as well as alternative methods for isolating islets [7-11]. The advantages of the CBD method described by Gotoh et al. are twofold: (1) perfusing the pancreas through the CBD allows collagenase to access the islets using anatomical structures, and (2) stationary digestion reduces mechanical damage to the islet. A comparison of these two methods showed that either method can be used to procure viable and functional rat islets; however, the Gotoh et al. method produced an islet yield approximately 50% higher and was more cost effective [12].

Our laboratory uses a modification of the CBD protocol when isolating rodent islets (*see ***Appendix A** for a annotated murine protocol and **Appendix B** for an abbreviated version). Although cannulation of the rodent bile duct requires technical skill, we are partial to this approach. Collagenase may interact more closely with the connective tissue surrounding the islets when delivered through intact anatomical structures, which results in a higher islet yield as suggested by others [12]. Szot et al. provide a detailed video account of a rodent islet isolation using a method of bile duct cannulation [13]. We describe an alternative method of injecting lobes of the pancreas in situ for cases when the bile duct has been compromised or cannot be used (*see ***Appendix A**, 12B). When evaluating any islet isolation protocol, one must consider that the outcome is influenced by differences in the type and concentration of collagenase used, the method of collagenase administration, the temperature and duration of pancreas digestion, the method for purifying islets from pancreatic acinar tissue, and the culture conditions following isolation.

### 2.2. Considerations for choosing collagenase

Collagenase enzymes are routinely used in digesting connective tissue that binds islets to other pancreatic tissue. Variability exists between manufacturers and between each lot of collagenase product from the same manufacturer. Enzyme activity, purity, and formulation, therefore, strongly influence the outcome of the islet isolation. The composition of collagenase and other enzymes in each lot must be ideal to the task of isolating islets specifically. Wolters et al. have described, in detail, the differences in isolation with purified collagenase types 1 and 2 separately, and the combination that yields the most effective rat islet isolation [14,15]. De Haan et al. formulated criteria for evaluating each lot of commercially available collagenase to ensure proper digestion of rat islets [16]. Formulations with increased collagenase activity, a specific range of both neutral proteases and clostripain, and with low levels of trypsin activity may produce the most viable islets [14,16]. We have found that optimal collagenase formulations for rat islet isolations also provide acceptable criteria for rating collagenase used in our mouse islet isolation procedures.

Digestive enzyme formulations for islet isolation range from crude collagenases to highly purified combinations used extensively in human islet transplants. Brandhorst et al. suggest that the differences from lot to lot may be due to the lack of proper accounting of tryptic-like activity, even in the most pure mixtures of collagenases, such as Liberase HI (Roche, Indianapolis, IN, USA) and collagenase NB1 (Serva, Heidelberg, Germany) used in isolation of human islets for transplantation [17]. The tryptic-like activity in enzyme blends may work in concert with the other enzymes to increase the activity of the digestion, although there is some debate about the damage tryptic-like activity has on the islets [18]. Enzyme blends with high purity and precise notation of the components are used in human islet isolation to ensure consistent activity and reproducibility [17,19]. Enzymes with higher purity, and consequently a higher price, are also used in human pancreatic islet isolation to reduce incidence of contamination by endotoxins.

### 2.3. Endotoxins are another consideration

Endotoxins correlate with increased proinflammatory cytokines in models of transplantation [19]. Endotoxins have been identified in both the digestion and gradient separation steps. Collagenase formulations as well as different types of gradient compounds have been identified as containing endotoxins in varying amounts [19]. Endotoxins are of particular concern in human-to-human islet transplantation procedures. Infiltration of transplanted islets by inflammatory cytokines has been attributed to endotoxin contamination [19].

### 2.4. The process of collagenase digestion

Almost as important as the specific formulation of collagenase is ensuring that a protocol is optimized to the collagenase type and activity and to the animal species and strain in a given protocol. Factors that influence the process include digestion time, digestion temperature, collagenase concentration, and the route of administration of collagenase, which vary widely among protocols. Perfusing the pancreas through the common bile duct allows collagenase to access the islets using biological structures, which may change the duration of digestion when compared to other methods. Differences between collagenase batches and other factors that influence digestion facilitate the need for testing each protocol for optimal islet viability and islet function, which remains paramount to success and reproducibility of islet isolation.

Our laboratory follows published guidelines for collagenase enzyme formulations [16], which provide consistency for expected outcomes during digestion. We use Collagenase P (Roche, Indianapolis, IN, USA) enzyme at 1.4 mg/mL in a modified Hank's Balanced Salt Solution (HBSS; Invitrogen, Carlsbad, CA, USA) injected into the pancreas via the CBD. The pancreas is then excised whole and placed in modified HBSS for stationary digestion at 37°C for 8–11 min as described in detail in **Appendices A** and **B**.

### 2.5. Gradient separation of islets and pancreatic acinar tissue

There is some debate regarding the use of a density gradient to purify islets from acinar tissue. Purifying islets from acinar tissue, regardless of the method, is important due to the nature of the pancreatic tissue. The cells of the exocrine pancreas secrete various digestive enzymes necessitating the separation of islets from pancreatic acinar tissue [20]. Sodium diatrizoate, Histopaque (Sigma-Aldrich, St. Louis, MO, USA), is a hypertonic solution also used in isolating other cell types. Our laboratory has used Ficoll 400 (Sigma-Aldrich, St. Louis, MO, USA) and Histopaque at different densities. In isolations with Ficoll 400 layered in a discontinuous gradient of 1.109, 1.096, 1.070, and 0.570 g/mL islets were isolated from the interfaces of both the 1.070/1.096 and 1.096/1.109 g/mL layers. However, we found that the preparations were often contaminated with acinar tissue. Our purity results generally improved using aseptically filled and premixed Histopaque, which is also available in sterile preparations. Combining Histopaque 1.119 g/mL with the 1.077 g/mL preparation to produce a 1.100 g/mL gradient appears to enhance islet purity. It should be noted that these studies comparing Histopaque and Ficoll were not rigorous, and both gradients are widely used and accepted. We provide our anecdotal evidence for consideration in choosing a purification method.

The final purity of the product depends on the animal strain and the characteristics of density gradients. In our experience, lean rodents tend to yield a higher purity of the final preparation than those with more fat. There is also a strain-dependent difference in the outcome of the gradient purification, which is consistent with findings describing strain-dependent differences in islet isolation [21].

A second purification of islets from acinar tissue is often needed to further increase islet purity prior to culture. Our protocol includes using a microscope to identify islets, then handpicking those islets from one suspension culture dish into a second dish containing a buffered solution or culture medium, such as HBSS or RPMI, respectively. Islets can then be transferred to a dish containing culture media for overnight incubation. Once islets have been transferred to media, minimizing time outside the sterile incubator will limit exposure to contamination and pH changes while handpicking islets for experiments.

### 2.6. Islet yield

The total number of islets found in a rodent pancreas varies considerably among strains. Bock et al. compared seven different mouse strains and found the number of islets per pancreas ranged from 971 ± 88 (129S6 mice) to 2,509 ± 133 (B6 mice) [22]. Using a mouse model of diabetes, Bock et al. identified a similar number of islets per pancreas (~3,200) for both ob/ob and ob/+ control mice; the islets from the diabetes-prone ob/ob mice were 3.6 times larger, however, than ob/+ controls [23]. In young male Wistar rats, Inuwa et al. demonstrated that the total islet number increased with age, ranging from ~6,000 to ~20,000 islets per pancreas [24]. Other studies have estimated the number of rat islets as low as approximately 3,000–5,000 per pancreas [25,26]. Therefore, the expected islet yield from an isolation procedure depends a great deal on the age and strain of the rodent.

The expertise of the technician, as well as the method of isolation chosen, will also influence the total islet yield. Consequently, a definitive expected yield is difficult to quantify. With an experienced technician yields from various mouse strains should range from 200–400 with average yields of 300 islets per mouse [6,9]. Rat yields range from approximately 600–800 islets per animal [21]. Based on estimates of total islet numbers, this suggests that the islet yield ranges from 10% to 30% for the typical rodent pancreas. For comparison, the human pancreas is thought to contain over one million islets, and the typical isolation yields approximately 250,000–450,000 islets as estimated by islet equivalents [27].

## 3. Islet culture conditions

After performing islet isolation, proper culture conditions are imperative to ensuring that the islets are able to recover from the insult of collagenase digestion. Examination of media with different glucose concentrations indicated that islets cultured with 11 mM glucose have lower apoptosis rates and increased viability than those in media with both higher and lower glucose concentrations for rodents [28]. Media with glucose concentrations substantially below 11 mM can reduce islet insulin content and downregulate key genes related to glucose metabolism, whereas extended exposure to high glucose causes toxicity [28,29]. Studies of optimal culture conditions demonstrated that RPMI 1640 with serum maintains or augments glucose-stimulated insulin secretion in murine islets [30]. Insulin secretion remained lower in islets cultured in five other types of culture media brought to comparable glucose concentrations [30]. Thus, properties apart from its higher glucose concentration (11 mmol/L) make RPMI 1640 suited for studies of insulin secretion in murine islets [30]. In another study CMRL1066, rather than RPMI 1640, was used to culture rat islets in order to preserve the insulin secretory capacity [16].

We use RPMI 1640 culture medium both for culturing islets and while purifying islets from acinar tissue after the density gradient separation. RPMI1640 is supplemented with 10% (*v*/*v*) fetal bovine serum to promote viability and with 100 U/mL penicillin and 100 μg/mL streptomycin to reduce the possibility of contamination. Islets are plated in 100 × 20 mm suspension culture dishes (Corning Inc., # 430591), rather than culture-treated dishes, to decrease islet attachment. Islets are cultured in 10 mL of RPMI 1640 media in these dishes.

To maintain islets for long-term culture, the optimal islet density is four islets per square centimeter in order to prevent competition for nutrients [16]. Overnight incubation of 16–20 h provides islets time to recover from the harsh process of collagenase digestion. Recovery in a sterile incubator at 37°C with 5% CO_2_ infusion and humidified air is necessary for islet function prior to performing viability or functional assessment assays [30]. Rodent islets can maintain glucose sensitivity for at least 1 week in culture with frequent medium changes [30] and perhaps even longer based on data from human islets [30,31]; however, changes in rodent islet function can occur between as little as between 1 and 4 days in culture [32].

## 4. Assessment of islet health and function

### 4.1. Morphology

**Figure 1:**
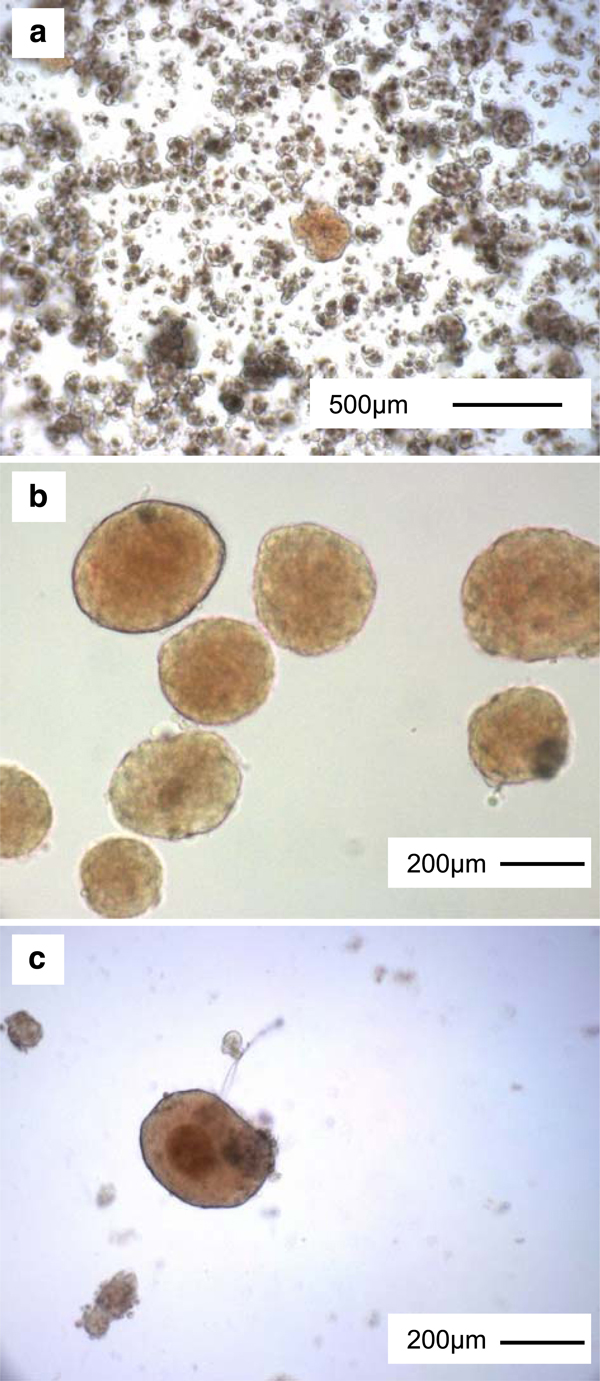
**Islet morphology following isolation**. **a** A freshly isolated islet shown among pancreatic acinar tissue on the day of isolation. **b** Islets purified from acinar tissue, after incubation at 37°C and 5% CO_2_ for 18–20 h. **c** Islet incubated 18–20 h with darkened, hypoxic center.

**Figure 2:**
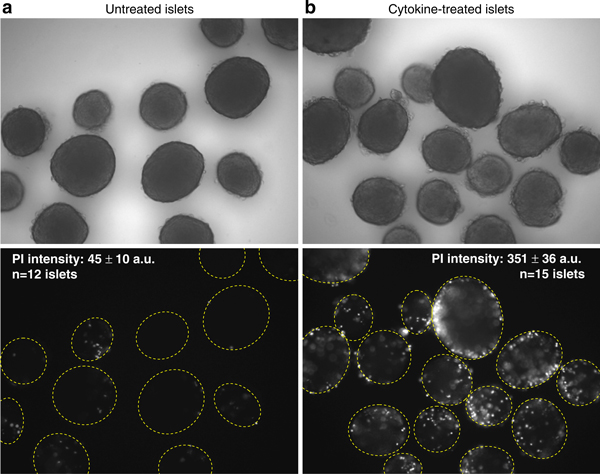
**Assessment of islet viability by fluorescence intensity of propidium iodide**. **a**, **b** Untreated (healthy) islets shown in brightfield (**a**, *top panel*) display only trace amounts of PI fluorescence (**a**, *bottom panel*). Islets treated overnight with a combination of cytokines (100 pg/mL TNF-α, 50 pg/mL IL-1β, 100 pg/mL IFN-γ) shown in brightfield (**b**, *top panel*), in contrast, display substantial PI fluorescence (**b**, *bottom panel*).

Visual inspection of the islets can provide some rudimentary information regarding health. When viewed with a scanning objective lens under a light microscope, islets appear spherical and golden-brown, approximately 50–250 μm in diameter. These features, particularly the darker color of islets in comparison to the relatively transparent exocrine tissue, allow for rapid identification of islets as shown in Figure 1a. Healthy isolated islets following overnight recovery also have few, if any, individual cells protruding from the relatively smooth rounded surface (Figure 1b). Cells protruding from the surface can be a sign of decreased or decreasing health (compare Figure 2a and 2b described below). Larger islets are also prone to developing hypoxic cells in their center, visibly distinguishable as darker cells compared to the surrounding tissue (Figure 1c). Reducing the amount of media in the dish to allow increased oxygenation may reduce this effect [33].

### 4.2. Islet viability

Supplementing visual inspection with additional techniques can provide quantification of islet viability and functionality. We define viability as living versus dead or dying cells as assessed using cell exclusion or DNA-binding dyes. A common approach in the human islet transplant field is to measure the ratio of healthy living cells to dead cells within each islet with fluorescence microscopy. Fluorescein diacetate (FDA) incorporates into healthy cells by facilitated diffusion and fluoresces blue; propidium iodide (PI; Sigma-Aldrich, St. Louis, MO, USA) is a membrane impermeant red fluorescent dye that is excluded from viable cells and enters only dead or dying cells [34]. Using these fluorescent dyes in combination, the health of islets can be assessed by the FDA/PI ratio. Unmanipulated isolated islets from healthy control animals generally have 90–95% viability, meaning blue FDA staining in 90–95% of the component cells of an islet and red PI staining is detectable in only 5–10% of the cells in any given islet. Additional techniques commonly used to measure islet viability include AnnexinV, SYTO-13/ethidium bromide, calcein AM/ethidium homodimer, fluorescein diacetate, and ethidium bromide which are more expensive but are also more sensitive to islet cell damage than FDA/PI [34-36].

We use a variation of the above approach by quantifying the mean intensity of PI fluorescence within an islet. Imaging software records the fluorescence intensity within a region of interest (ROI) designated by an encircled islet. Healthy islets typically display smooth round borders (Figure 2a, top panel) and show minimal PI staining (Figure 2a, bottom panel). Unhealthy cells, such as islets cultured overnight in a mixture of proinflammatory cytokines (Figure 2b), exhibit cells protruding from the rough islet surface (top panel) and significantly greater PI staining (bottom panel). The mean pixel fluorescence intensity of the ROIs is used to quantify cell death as listed atop the lower panels of Figure 2a, b.

### 4.3. Glucose-stimulated insulin secretion

A fundamental property of pancreatic islets is their capacity to regulate the release of insulin and other hormones in direct response to changes in extracellular glucose concentration. In large part, this ability defines islet function since insulin is produced and released in the body only from islet beta cells. Insulin is crucial to regulating blood glucose, and reduced insulin secretion is a key feature of both Type 1 and Type 2 diabetes [1]. Glucagon, somatostatin, and other peptides are also produced by islet cells, but these are secreted in smaller amounts and more difficult to detect. Glucose-stimulated insulin secretion (GSIS) is thus a well-accepted measure of islet function.

To measure GSIS, islets are cultured in a 'low' glucose concentration, typically near 3 mM, to measure the amount of insulin secreted into the media under 'basal' or 'unstimulated' conditions. Stimulated insulin release is measured by exposing islets to a higher glucose concentration such as 11.1 mM (half-maximal) or >28 mM (maximal). In response to the glucose stimulation, the time course of the islet response is biphasic, consisting of a rapid spike in insulin release (first phase) followed by a decline to a prolonged second phase plateau of insulin that remains throughout the duration of the stimulus. GSIS can be measured either by static conditions or by perfusing islets to measure the kinetics of insulin release in response to glucose. Each technique has its advantages and disadvantages, which are reviewed elsewhere [37]. The glucose stimulation index (SI) is the ratio of stimulated-to-basal insulin secretion. Healthy islets have an SI of 2–20 depending on several factors including strain, age, and body weight.

### 4.4. Glucose-stimulated calcium

In nearly any cell type, the tight regulation of intracellular calcium ([Ca^2+^]_i_) is crucial to normal functioning of many cellular processes including metabolism, signal transduction, and exocytosis [38]. As in the GSIS assay, analyzing islet [Ca^2+^]_i_ in response to changes in extracellular glucose concentration provides supportive information about islet viability and function because calcium is an integral component of the insulin secretion pathway [39-41]. Therefore, measuring the glucose-stimulated [Ca^2+^]_i_ response (GSCa) in islets offers a reasonable reflection of insulin secretion [42] and more importantly, a potentially sensitive indicator of overall islet health and function in vitro [43]. A number of fluorescent probes are commonly used to measure intracellular calcium including fura-2 acetoxy-methyl-ester (fura-2AM), fluo-3, fluo-4, and fura red.

**Figure 3:**
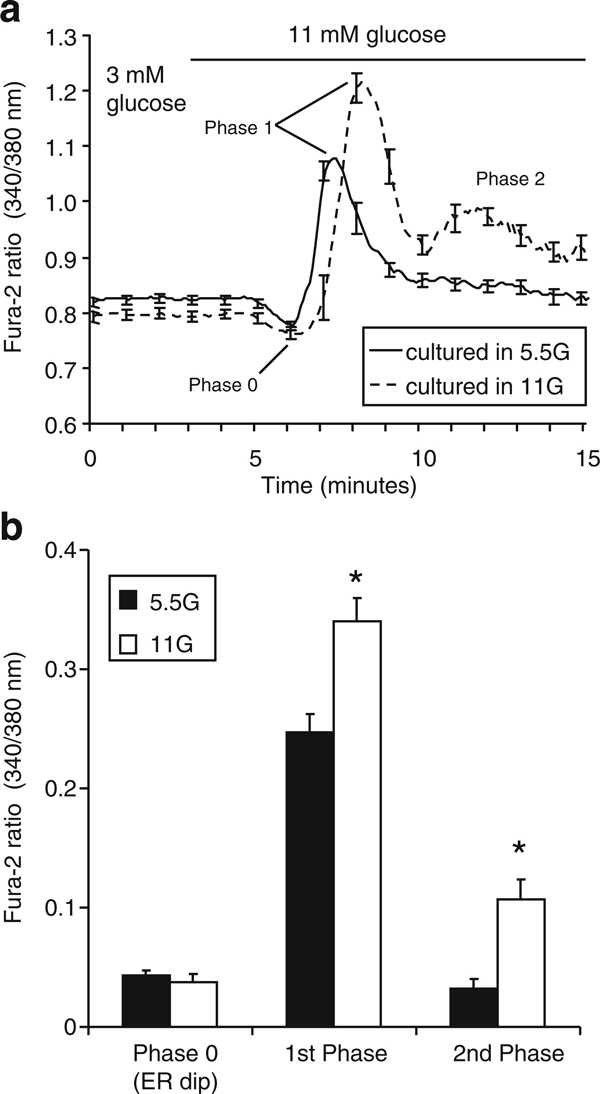
**A comparison of glucose-stimulated calcium (GSCa) response for islets cultured overnight in 5.5 mM glucose (5 G) and 11 mM glucose (11 G)**. **a** GSCa for islets cultured in 5.5 mM glucose (*solid*, mean of *n* = 8) and 11 mM glucose (*dashed*, mean of *n* = 9). *Arrows* indicate the three phases of GSCa; stimulation with 11 mM glucose is indicated by the *horizontal bar*. **b** Mean amplitude of the calcium response during each phase (phase 0, 1, and 2) for islets cultured in 5.5 mM glucose (solid, *n* = 37) or 11 mM glucose (stripes, *n* = 34); * indicates *p* < 0.05. Note that although a difference in latency is apparent in (**a**), this not a consistent or significant effect in the larger data set.

As a demonstration of GSCa, we compared islets cultured overnight in RPMI 1640 containing either 5.5 mM glucose or 11 mM glucose. Following overnight culture treatment, islets were loaded with fura-2AM [43], and then [Ca^2+^]_i_ was recorded in Krebs-Ringer-bicarbonate containing 3 mM glucose followed by stimulation with 11 mM glucose. The normal GSCa consists of three phases: phase 0 (the initial dip below baseline due calcium uptake by the endoplasmic reticulum), phase 1 (the rapid rise to peak calcium that is concomitant with the release of pre-docked insulin granules), and phase 2 (the elevated plateau; [44]. As shown in Figure 3a, the GSCa was more robust for islets cultured in 11 mM glucose RPMI 1640 compared to islets cultured in 5.5 mM glucose RPMI 1640. Specifically, the mean amplitude (calculated as the change in calcium from basal levels) of phase 1 and phase 2 was significantly higher among islets cultured in 11 mM glucose (Figure 3b). These differences are consistent with previous reports of optimal viability and function when islets are cultured in 11 mM glucose culture media [28,30] and demonstrate the sensitivity of using GSCa as a valuable technique for assessing islet function.

There are several advantages of measuring GSCa over GSIS. Substantial time and costs are involved in GSIS since insulin must be measured by immunoassay following the GSIS experiment, whereas GSCa provides results in real-time without additional expense. Furthermore, GSCa utilizes frequent sampling, which allows for precise temporal analysis of the amplitude, latency, and trajectory of changes in response to glucose stimulation. Also, GSCa can be used to assess individual islets, so fewer than ten islets would be sufficient to characterize the function for the entire batch. In contrast, islets must be grouped together for GSIS to produce detectable quantities of insulin, especially for islet perfusion studies (we use 50 islets for each replicate in perfusion studies). One limitation, however, is that GSCa cannot be easily normalized to a standard value in the same way that insulin is normalized to a standard set of insulin concentrations. Coupled with potential differences in dye loading and changes in light source intensity/efficiency over long periods of time, these issues make batch-to-batch comparisons of different islet preparations using GSCa difficult. These issues can be somewhat mitigated by determining the stimulation index, which is commonly used to assess GSIS from donor to donor in human islets for transplantation purposes [45].

### 4.5. Islet dissociation and cell identification

Islets are composed of several distinct cell types consisting of the glucogon-secreting alpha cells, insulin-secreting beta cells, somatostatin-producing delta cells, and others [46-48]. The percentage of these cells, as well as their anatomical locations in islets, varies between species. In rodents, the majority of cells are beta cells (65–85%) and alpha cells (10–25%), with the remaining 5–10% of cells consisting of delta cells and other cell types [46-48]. Isolating these cells requires either mechanically disrupting the bonds between cells or using a digestive enzyme to separate the cells [49]. Once islets have been separated into their component cells, use of counterflow elution as described by Pipeleers [50], or light scatter flow cytometry as described by Rabinovitch et al. [51] may be used to identify and purify the cells. We provide a detailed protocol describing the dissociation and culturing of murine islet cells in Appendix **C**.

## 5. Conclusions

The ability to consistently procure viable and functional islets is crucial to effectively studying the physiology and pathophysiology of islets and their constituent cells. As stated previously, islet isolation is an intricate process. In this review, we have addressed the key factors to consider in the isolation and assessment processes to obtain both viable and functional islets. In the accompanying protocol, we provide a method developed by integrating the reports of many others in the research field with careful experimentation to optimize the islet isolation process for our laboratory. While following this protocol provides a start for islet isolation, any procedure must be optimized to the capabilities of the laboratory and the specific goals of the study.

## Appendix A: Isolation of islets from mice

### Materials

Two forceps

One Fine Iris scissors for internal use

One standard pattern scissors

One to two Bulldog clamp(s)

30 or 27 Ga 1/2" needle

5 mL Luer-lock syringe

10 mL pipette

15 mL conicals

50 mL conicals

2" × 2" gauze

37°C water bath

Scale (accurately measures in milligrams)

Centrifuge

### Solutions

For anesthesia:

CO_2_ gas

  or

Solution (A): Mix 2 mL of ketamine and 1 mL of xylazine

Ketamine (60–80 mg/kg) and xylazine (10 mg/kg)

Solution (B): Mix (A) and 7 mL of Normal Saline, injection.

Various methods of anesthesia are used to ensure that research protocols follow humane methods. The following are used by our laboratory: CO_2_ gas for euthanasia, or Ketamine/Xylazine for IP injection, followed by servical dislocation once animals are in deep anesthesia. With the ketamine/xylazine, animals are given 0.005 mL per 1 g body weight to induce anesthesia, followed by cervical dislocation to induce death.

For islet isolation:

G-Solution sterile filtered in 0.22 μm filter:

HBSS (Invitrogen #14065-056 diluted to 1×)

0.35 g NaHCO_3_/L

1% bovine serum albumin (BSA)

This solution, as with any cell culture solution, must be sterile filtered. This method is performed using clean techniques, not sterile techniques; therefore, it is vital that the solutions are sterile prior to use.

Other protocols use protease inhibitors or varying concentrations of BSA to inactivate endogenous proteases, some as high as 10% BSA.

Collagenase solution:

Solution (C): 1.4 mg/mL Collagenase-P (Roche #1129 002 001) in G-Solution (prepare 5 mL/pancreas)

Collagenase is an area where there are differences in protocols. We have chosen to maintain a constant concentration of 1.4 mg/mL and to vary the digestion time to suit different lot numbers. Studies use different collagenase types, but we ensure specific criteria are met by each lot prior to use. The digestion time is based on islet function and viability experiments before using a new enzyme lot experimentally.

Gradient:

Histopaque 1100 Solution (1.100 g/mL):

100 mL Histopaque 1077 (SIGMA # 10771)

120 mL Histopaque 1119 (SIGMA # 11191)

We prepare and store a stock solution at 4°C. Only the amount of Histopaque required is brought to room temperature prior to its use. It is also important to note that the Histopaque solutions should be kept in the dark.

Culture media:

RPMI 1640 + L-Glutamine (Gibco #11875-093)

10% FBS (Gibco #16000-044)

Penicillin (100 U/mL)/streptomycin (100 μg/mL; Gibco # 15140-122)

### Protocol

1. Prepare 1 mL of G-solution for each mouse in a 15 mL conical.

2. Place each 15 mL conical from **Step 1** in ice.

In order to inhibit the collagenase digestion prior to incubation, place a 15 mL conical containing 1 mL of HBSS for each mouse on ice. The ice will maintain the collagenase-filled pancreata at a temperature which inhibits digestion. The ice also serves to maintain uniform conditions while isolating islets from multiple pancreata.

3. Euthanize animal with CO_2_ or inject mouse IP with 0.01 mL/g body weight of solution (B). The mouse is ready for exsanguination after no response to pinching its foot. Note: the investigator MUST refer to institution Animal Care and Use Committee guidelines and policies regarding proper procedures when handling and using animals in research.

Some researchers prefer to use ketamine/xylazine when collecting blood samples.

**Figure 4:**
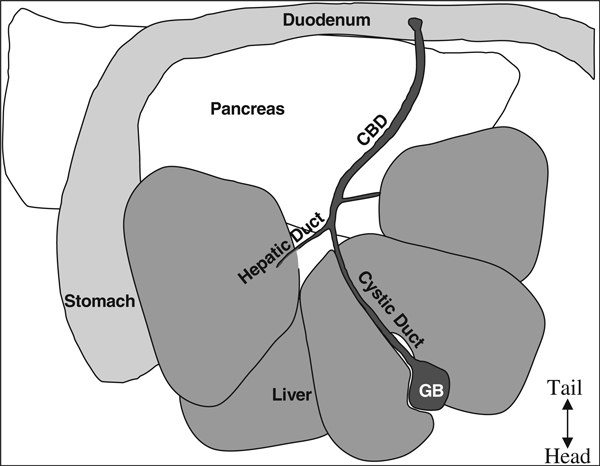
**Anatomy of the mouse upper intraperitoneal (IP) cavity**. NOTE: The top is the caudal portion of the mouse, bottom is the rostral portion, ie, this is from the perspective of the mouse lying on its back tail away from the surgeon and nose toward the surgeon.

4. (Optional) Exsanguinate the animal by heart perfusion using a 1 mL syringe with 25 Ga needle.

5. Wet abdominal fur with 70% alcohol to reduce the chance of hair contamination in the IP cavity during subsequent steps.

6. Open abdomen with standard pattern scissors in a V-shape starting from the lower abdomen and extending to the lateral portions of the diaphragm in order to expose all organs in the peritoneal cavity (Figure 4).

7. Turn the animal so that the nose is closest to the surgeon and the tail points away from the surgeon.

This position allows for cannulation into the common bile duct from the rostral end closest to the liver at the junction of the cystic and hepatic ducts, leaving space to inject again closer to the duodenum if an error occurs.

8. Secure the liver with 2" × 2" gauze.

Lay the liver flat and place an unfolded 2" × 2" piece of gauze under the liver closest to the surgeon. Gently flip the liver over onto the gauze exposing the junction of the gall bladder and common bile duct. Fold the remaining gauze over the liver to secure it during cannulation of the common bile duct.

9. Using a Johns Hopkins Bulldog clamp (Roboz# RS-7441), clamp off the common bile duct near the junction with the small intestine. Alternatively, tightly tied suture string can be used to tie off the CBD at the junction with the small intestine.

**Figure 5:**
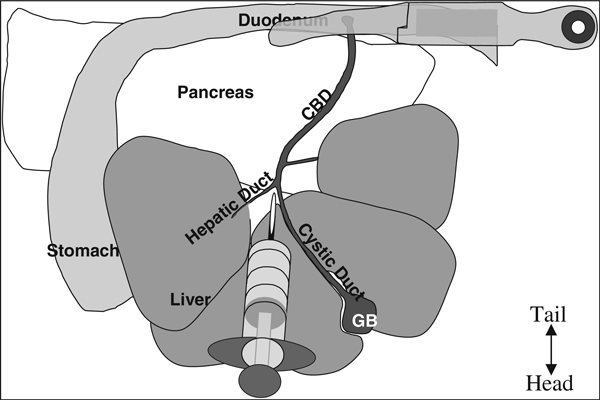
**Injection site and clamping the Common Bile Duct (CBD) at the duodenum**.

The pancreas contains a system of ducts that allows pancreatic enzymes to flow into the gut. The pancreatic duct merges with the inferior end of the CBD where the pancreatic enzymes enter the duodenum (*see* Figure 4). This pancreatic duct provides the most readily available access to the endocrine pancreas. Clamp the CBD as close to the junction with the small intestine as possible so as not to occlude the pancreatic duct (*see* Figure 5). Clamping the duodenum on either side of the junction with the CBD is also an option. Using a clamp allows access to the pancreatic duct while closing off access to the duodenum. Also attached to the CBD are several hepatic ducts. After becoming proficient with the perfusion technique, the major hepatic duct that leads to the caudal right lobe of the liver could also be clamped to ensure that minimal collagenase is delivered to the liver.

10. Fill a 5 mL syringe with solution (C).

11. Cannulate the CBD with a 27–30 gauge 0.5 in. needle secured to a 5 mL syringe or a 27–30 gauge butterfly needle. Cannulate the CBD at the junction of the cystic duct from the gall bladder and left hepatic duct from the liver (forms a Y-shape, *see* Figure 5).

At the most superior portion of the CBD the junction of the cystic duct and hepatic duct forms a Y-shaped reference point in most mice. This location allows the surgeon the opportunity to cannulate the CBD with a 27 gauge butterfly needle or a 27 gauge 0.5 in. needle in 5 mL syringe. Tips for a successful cannulation of the duct: (1) use a new needle for each attempt because the needle may dull very easily; (2) place the needle at the Y-shaped junction and slide the needle into the duct moving in 1 mm increments (small-scale movement is the key to success); (3) if the duct is difficult to visualize, squeeze gently on the gall bladder which may cause the CBD to turn yellow as bile flows through.

12. Inject 4–5 mL of solution (C) into CBD.

A. An injection of 4–5 mL collagenase solution into the CBD should be sufficient to completely inflate the pancreas such that there is fluid in all regions of the pancreas from head to tail. Ensuring that the collagenase inflates the pancreas in its entirety allows for the maximum yield of islets. The distribution of islets in the pancreas is not uniform; studies show a higher concentration of islets in the pancreatic tail, which makes the full inflation of the pancreas even more important. This step requires skill of the surgeon to obtain consistent results, and success is directly related to islet yield.

B. Failed attempts to infuse the pancreas with collagenase through the hepatic duct eventually destroy the duct. An alternative method involves directly injecting collogenase into the numerous lobes of the pancreas. In this method, it is important to inject small amounts (about one to two drops) of collagenase into as many of the lobes of the pancreas as possible. Begin by locating and lifting the spleen ~1 in. above the body cavity with forceps. The pancreas is attached to the spleen and should be visible. Beginning at the top, inject two to three drops of collagenase to inflate individual lobes of the pancreas using a 5 mL syringe with 30 gauge needle. As the lobe inflates, move the needle downward into the next lobe and the next, allowing gravity to help get collagenase into the pancreas, until the needle hilt reaches the tissue (~1 cm). Remove the needle, and reenter the tissue at another section of tissue to inflate other sections. Adjust forceps as necessary to hold new sections of pancreas above the body cavity in order to inject downward. Continue to follow the pancreas with the needle and inject the pancreatic lobes along the stomach and intestine. When as many lobes have been injected as possible, remove the pancreas and continue the protocol.

13. Remove pancreas from mouse and place into a 15 mL conical from **Step 1**.

A. During the removal of the pancreas, it is important to avoid intestinal rupture in order to reduce the risk of contamination of the cell culture by intestinal bacteria. Removal begins at the point of attachment of the CBD to the duodenum. Using forceps or scissors snip the CBD and gently detach the pancreas from the intestines. Then, cut the CBD from the attachment to the liver. Continue to remove the pancreas from the greater curvature of the stomach and finally the spleen. Place the removed pancreas into the 15 mL conical that holds 1 mL of cold HBSS and replace the tube in the ice.

B. After injecting the pancreas, place the pancreas that has been directly injected with collagenase into 1 mL of collagenase solution on ice, rather than HBSS, as in the preferred CBD-cannulation method above.

14. Repeat the above steps for all mice, then proceed to the next step.

Follow the above procedure for the remaining animals placing one pancreas per tube on ice.

15. Incubate for *8 min at 37°C in 15 mL conical tube. *Incubation time varies among different lots of collagenase (usually 8–11 min).

After collecting the pancreata into their respective tubes on ice, place the same tubes directly into a water bath at 37°C to incubate for 8–11 min. The incubation time varies between lots of collagenase; therefore, it is important to test each lot prior to use in experimental conditions.

16. Shake the 15 mL tube containing digested pancreas hard by hand for approximately 5 s to complete the separation of tissue (the result should be the consistency of pea soup and free of large pieces of pancreatic tissue if the pancreas was completely inflated).

This step results in the mechanical digestion of the pancreas and can be performed for all tubes simultaneously if the tubes are placed into a rack for incubation and covered while shaking. If fat tissue was incubated with the pancreas, it will remain undigested after shaking.

17. Quickly place the 15 mL conical in ice and fill to 15 mL with G-solution to dilute collagenase concentration and slow the digestive process.

18. Centrifuge 2 min at 1,200 rpm (290×*g*).

19. Discard the supernatant by decanting into a waste container.

20. Wash with of 10 mL G-Solution, 2 min at 1,200 rpm (290×*g*).

All wash steps, unless otherwise stated, involve resuspending the tissue pellet with a sterile 10-mL pipette using room temperature HBSS followed by room temperature centrifugation at 290×*g* for 2 min.

21. Discard the supernatant by decanting into a waste container.

22. Add 10 mL of G-solution and resuspend with a 10 mL pipette.

23. Filter each sample through a size 40 (420 μm) sieve (Bellco Glass, Inc, Cat# 1985-00040) into separate 50 mL conicals.

It is important to put each pancreas into its own conical so that the gradient can sustain the tissue load and properly separate the islets from the exocrine tissue. Overloading the gradient will lead to reduced islet yield and purity.

24. Bring volume to 20 mL with G-solution.

25. Centrifuge at 1,200 rpm for 2 min to pellet resuspended tissue.

26. Decant supernatant, and place the opening of the 50 mL conical onto a paper towel to remove as much liquid as possible.

Removing all of the liquid ensures that the gradient density is not diluted when the pellet is resuspended in the gradient.

27. Resuspend pellet in 10 mL Histopaque 1100 Solution with a 10 mL pipette.

Resuspending the tissue completely in the gradient ensures that islet density is not affected by attached exocrine tissue. Tissue filtered through the sieve should resuspend to a state that appears almost homogenous in the gradient.

28. Centrifuge for 20 min at 1,200 rpm. Note: islets are in the supernatant.

After centrifugation, islets should be visible in the supernatant of the gradient as floating white specks. If there are fewer islets present than expected, resuspend the pellet produced during the previous step in order to inspect for islets. Also note that if more than one pancreas is added to the gradient, many islets will be found in the pellet. This should not be the case if the gradient contains only one mouse pancreas.

29. Label a new 50 mL conical, and add 25 mL of G-solution.

30. Decant the supernatant into the new 50 mL conical with G-solution from the previous step.

Note: collect all washes below to be able to look through them later.

31. Centrifuge at 1,500 rpm (453×*g*) for 4 min and decant the supernatant. Note: islets are in the pellet.

Take care to pour the solution into the waste container in one motion in order to avoid having the solution wash back onto the pellet causing the islets to become dislodged and subsequently decanted.

32. Add 10 mL of G-solution to the pellet, and pipette up and down several times with a 10 mL pipette.

The final washes of the pellet are very important to ensuring that the islets are clear of the Histopaque solution and that the islets are well separated from one another prior to plating in culture dishes.

33. Centrifuge at 1,200 rpm for 3 min.

34. Decant the supernatant and replace with 10 mL of culture medium.

RPMI1640 with 10%FBS and Pen/Strep

35. Transfer to a sterile Petri dish under culture hood to incubate or pick/clean islets under a dissecting or light microscope.

Use a suspension (Petri) culture dish so that the islets do not stick as they would in a tissue culture treated. Incubate or pick clean islets under dissecting or light microscope using low-retention pipette tips.

## Appendix B: Isolation of islets from mice

### Materials

Two forceps

One Fine Iris scissors for internal use

One standard pattern scissors

One to two Bulldog clamp(s)

30 or 27 Ga 1/2" needle

5 mL Luer-lock syringe

10 mL pipette

15 mL conicals

50 mL conicals

2" × 2" gauze

37°C water bath

Scale (accurately measures in milligrams)

Centrifuge

### Solutions

For anesthesia:

CO_2_ gas

  or

Solution (A): Mix 2 mL of ketamine and 1 mL of xylazine

The concentration of xylazine used is 20 mg/mL.

Solution (B): Mix (A) and 7 mL of Normal Saline, inj.

The dosage of ketamine/xylazine used to induce anesthesia is typically 0.005 mL per gram of body weight. Euthanasia is induced by doubling this volume. (A 20 g mouse would receive 0.1 mL of drug for anesthesia, 0.2 mL for euthanasia) followed by cervical dislocation to ensure death.

For islet isolation:

G-Solution sterile filtered in 0.22 μm filter:

HBSS (Invitrogen Cat#14065-056 diluted to 1 × 0.35 g NaHCO_3_/L

1% bovine serum albumin

Collagenase solution:

Solution (C): 1.4 mg/mL Collagenase-P (Roche #1129 002 001) in G-Solution (prepare 5 mL/pancreas)

Gradient:

Histopaque 1100 solution (1.100 g/mL):

   100 mL Histopaque 1077 (SIGMA # 10771)

   120 mL Histopaque 1119 (SIGMA # 11191)

Culture media:

   RPMI 1640 + L-Glutamine (Gibco #11875-093)

   10% FBS (Gibco #16000-044)

   Penicillin (100 U/mL)/streptomycin (100 μg/mL; Gibco #15140-122)

### Protocol

**Figure 6:**
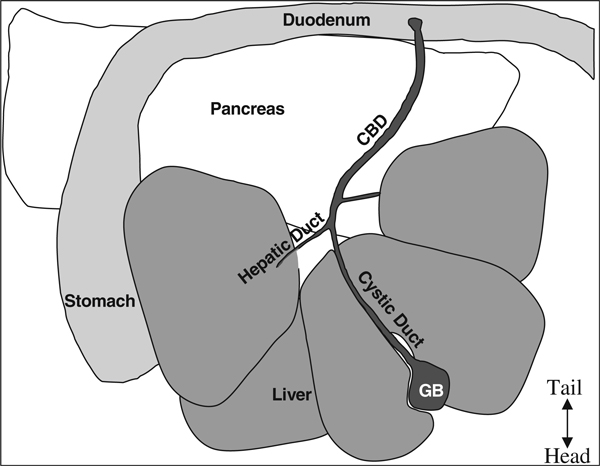
**Anatomy of the mouse upper intraperitoneal cavity**. The *top* is the caudal portion of the mouse, *bottom* is the rostral portion, i.e., this is from the perspective of the mouse lying on its back tail away from the surgeon and nose toward the surgeon.

**Figure 7:**
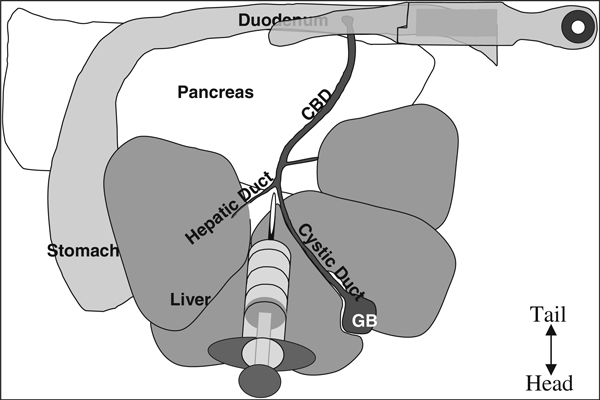
**Injection site and clamping the common bile duct (*CBD*) at the duodenum**.

1. Prepare 1 mL of G-solution for each mouse in a 15 mL conical.

2. Place each 15 mL conical from **Step 1** in ice.

3. Euthanize animal with CO_2_ or inject mouse IP with 0.01 mL/g body weight of solution (B). The mouse is ready for exsanguination after no response to pinching its foot. Note: the investigator must refer to institution Animal Care and Use Committee guidelines and policies regarding proper procedures when handling and using animals in research.

4. (Optional) Exsanguinate the animal by heart perfusion using a 1 mL syringe with 25 Ga needle.

5. Wet abdominal fur with 70% alcohol to reduce the chance of hair contamination in the IP cavity during subsequent steps.

6. Open abdomen with standard pattern scissors in a V-shape starting from the lower abdomen and extending to the lateral portions of the diaphragm in order to expose all organs in the peritoneal cavity (Figure 6).

7. Turn the animal so that the nose is closest to the surgeon and the tail points away from the surgeon.

8. Secure the liver with 2" × 2" gauze.

9. Using a Johns Hopkins Bulldog clamp (Roboz# RS-7441), clamp off the common bile duct near the junction with the small intestine. Alternatively, tightly tied suture string can be used to tie off the CBD at the junction with the small intestine.

10. Fill a 5 mL syringe with solution (C).

11. Cannulate the CBD with a 27–30 gauge 0.5 in. needle secured to a 5 mL syringe or a 27–30 gauge butterfly needle. Cannulate the CBD at the junction of the cystic duct from the gall bladder and left hepatic duct from the liver (forms a Y-shape, *see* Figure 7).

12. Inject 4–5 mL of solution (C) into CBD.

13. Remove pancreas from mouse and place into a 15 mL conical from **Step 1**.

14. Repeat the above steps for all mice, then proceed to the next step.

15. Incubate for *8 min at 37°C in 15 mL conical tube. *Incubation time varies among different lots of collagenase (usually 8–11 min).

16. Shake the 15 mL tube containing digested pancreas hard by hand for approximately 5 s to complete the separation of tissue (the result should be the consistency of pea soup and free of large pieces of pancreatic tissue if the pancreas was completely inflated).

17. Quickly place the 15 mL conical in ice and fill to 15 mL with G-solution to dilute collagenase concentration and slow the digestive process.

18. Centrifuge 2 min at 1,200 rpm (290×*g*).

19. Discard the supernatant by decanting into a waste container.

20. Wash with of 10 mL G-Solution, 2 min at 1,200 rpm (290×*g*). Discard the supernatant by decanting into a waste container.

21. Add 10 mL of G-solution and resuspend with a 10 mL pipette.

22. Filter each sample through a size 40 (420 μm) sieve (Bellco Glass, Inc, Cat# 1985-00040) into separate 50 mL conicals.

23. Bring volume to 20 mL with G-solution.

24. Centrifuge at 1,200 rpm for 2 min to pellet resuspended tissue.

25. Decant supernatant, and place the opening of the 50 mL conical onto a paper towel to remove as much liquid as possible.

26. Resuspend pellet in 10 mL Histopaque 1100 Solution with a 10 mL pipette.

27. Centrifuge for 20 min at 1,200 rpm. Note: islets are in the supernatant.

28. Label a new 50 mL conical, and add 25 mL of G-solution.

29. Decant the supernatant into the new 50 mL conical with G-solution from the previous step.

Note: collect all washes below to be able to look through them later.

30. Centrifuge at 1,500 rpm (453×*g*) for 4 min and decant the supernatant. Note: islets are in the pellet.

31. Add 10 mL of G-solution to the pellet, and pipette up and down several times with a 10 mL pipette.

32. Centrifuge at 1,200 rpm for 3 min.

33. Decant the supernatant and replace with 10 mL of culture medium.

34. Transfer to a sterile Petri dish under culture hood to incubate or pick/clean islets under a dissecting or light microscope (Figure 7).

## Appendix C: dissociation and culturing of murine islet cells

### Materials

Glass specimen tube

Glass 5 3/4" pasture pipets

Glass cover slips

0.4% gel to coat cover slips

Six well, non-tissue-culture-treated sterile plate

Roboz Micro Dissecting 0.8 mm tip forceps

50 mL centrifuge tube

### Solutions

Culture medium:

RPMI 1640 + L-Glutamine (Gibco #11875-093)

10% FBS (Gibco #16000-044)

Penicillin (100 U/L)/streptomycin (100 μg/mL; Gibco #15140-122)

Dissociation buffer:

Trypsin EDTA solution (Gibco #15400-054)

This should contain 0.5 g/L of trypsin (1:250) and 0.2 g/L of EDTA × 4 Na in Hanks' Balanced Salt Solution without CaCl_2_, MgCl_2_ × 6H_2_O, and MgSO_4_ × 7H_2_O.

Silicon solution:

Sigmacote (Sigma #SL-2)

### Protocol

1. Autoclave microscope cover slips and the Roboz micro dissection forceps as well as all glassware to be used for the dissociation. Allow sufficient time to dry completely.

A. To autoclave the cover slips, use aluminum foil to make long, rectangular packet that can hold six or seven cover slips that overlap slightly (no more than 1/4 of the width/diameter of the slip). Place the packet into an autoclave pouch. To minimize the chance of breaking the cover slips, place no more than three packets into the autoclave pouch.

B. Wrap the Roboz micro dissection forceps in foil and place in a small autoclaveable pouch as well.

2. The day before the dispersion, isolate islets and allow recovery overnight in a 37°C incubator with 5% CO_2_. If the islets are received from elsewhere, allow recovery overnight in fresh medium as above.

3. Place one slip into a well of a six-well plate.

4. Coat the cover slips with a 0.4% gelatin coating. Allow to sit in the hood for 15 min, then aspirate the excess gelatin off the cover slides.

5. Place the coated cover slips in incubator overnight to dry. If rushed, 2 hours in an incubator should be sufficient to allow the gel coating to dry.

6. Aliquot RPMI 1640 growth medium into a 50 mL tube and warm. A minimum of 4 mL is needed for every 100 islets.

7. Aliquot 500 μL trypsin per 100 islets into a second 50 mL centrifuge tube and allow to warm to 37°C.

8. To keep islets and the cells from sticking to the tubes, coat glass specimen tubes and glass pasture pipets with Sigmacote (Sigma #SL-2), following Sigmacote protocol.

9. Place 500 μL of trypsin in Sigmacoted specimen tube. If the trypsin is cold, cover with parafilm and place in the incubator to warm.

10. Use 100 islets per specimen tube, the larger the islet, the better. Place islets with a minimum of medium, into the warm trypsin. Perform this step one tube at a time. Do not allow the islets to sit in the trypsin for prolonged periods of time.

11. Triturate the islets using a Sigmacoted pasture pipet. Triturate rapidly against the sides of the tube while trying to avoid massive amounts of bubbling, or suctioning into the pipettor.

12. Observe the islet/trypsin mix to see if you can still see the islets. If so, triturate again to try and break the islets apart.

13. Add 1.5 mL RPMI 1640 to each specimen tube to neutralize the action of the trypsin.

14. If doing more than one tube of islets, place parafilm over the top of the tube and place in the incubator. Proceed to the next tube repeating the steps above until all tubes are done.

15. Place the tubes containing the cell suspension in a centrifuge and spin at 800 rpm for 3 min.

16. Carefully remove the supernatant by aspirating. Locate the cell pellet at the bottom of the tube, and aspirate without disturbing the pellet. (The easiest way to aspirate is to carefully tilt the tube and insert the aspiration line. Tilt the tube in a smooth motion to avoid backwash that could disturb the cell pellet. Aspirate as much of the supernatant as possible.)

17. Resuspend the pellet in 500 μL of warm RPMI 1640. Use a 1,000 μL pipette to aliquot the RPMI into the tube and a Sigmacoted pasture pipet to resuspend.

18. Using either a siliconized or low sample retention 1,000 μL pipette tip to aliquot the cell suspension onto the gel-coated cover slips.

19. Place 250 μL of the suspension onto each gel coated coverslip. Note that the cells will concentrate into the center of the drop.

20. Place the plate into the incubator for 45 min to 1 h to allow the cells to settle. The drops of suspension will spread out to cover the slide while in the incubator.

21. After 45 min to 1 h, add 2 mL of fresh, pre-warmed RPMI 1640 culture medium to each well. Carefully add the medium by allowing it to gently flow down the side of the well, in order to minimize disturbances to the cells.

22. Place in the incubator and allow cells to recover overnight.

23. The following day warm 2 mL of RPMI 1640 culture medium per well.

24. Carefully aspirate one well at a time and add 2 mL of fresh medium, again along the side of the well to minimize agitating the cells. Work quickly to minimize exposure to air.

25. Check the cells under a microscope, remembering that the greatest concentration will be where the center of the drop or drops were placed.
